# Imaging and Molecular Biomarkers of PFAS-Related Vascular Aging: A Narrative Review

**DOI:** 10.3390/ijms27136064

**Published:** 2026-07-06

**Authors:** Andrea Borghini, Francesco Faita, Ludovica Simonini, Mariangela Palazzo, Cinzia Sagheddu, Chiara Cavigli, Gabriele Donzelli, Elisa Bustaffa, Stefano Masi, Francesca Gorini, Fabrizio Minichilli

**Affiliations:** 1 CNR Institute of Clinical Physiology, 56124 Pisa, Italy; francesco.faita@cnr.it (F.F.); ludovicasimonini@cnr.it (L.S.); mariangelapalazzo@cnr.it (M.P.); cinziasagheddu@cnr.it (C.S.); chiaracavigli@cnr.it (C.C.); gabrieledonzelli@cnr.it (G.D.); elisa.bustaffa@cnr.it (E.B.); francesca-gorini@cnr.it (F.G.); 2 Department of Clinical and Experimental Medicine, University of Pisa, 56126 Pisa, Italy; stefano.masi@unipi.it

**Keywords:** PFAS, vascular aging, atherosclerosis, imaging biomarkers, molecular biomarkers

## Abstract

Per- and polyfluoroalkyl substances (PFAS) are persistent environmental contaminants increasingly associated with cardiovascular disease. Identifying early manifestations of vascular aging before the onset of overt disease is essential for improving cardiovascular risk stratification and prevention. Emerging evidence suggests that PFAS exposure contributes to early vascular and atherosclerotic alterations detectable by imaging techniques, including increased carotid intima–media thickness (CIMT), arterial stiffness, and endothelial dysfunction. In contrast, evidence for associations with coronary artery calcium progression and coronary stenosis remains scarce. Mechanistically, PFAS exposure promotes endothelial dysfunction, oxidative stress, chronic inflammation, lipid dysregulation, and genetic and epigenetic modifications, all of which contribute to premature vascular aging and metabolic disturbances. The integration of imaging and molecular biomarkers may provide complementary insights into the structural, functional, and biological processes underlying PFAS-related vascular damage; however, to date, this field remains largely unexplored. This narrative review summarizes current evidence on imaging and molecular biomarkers of PFAS-induced vascular aging and discusses their potential role in cardiovascular risk assessment. It also highlights key knowledge gaps and the need for robust epidemiological and multi-omics studies to validate these biomarkers, clarify causal mechanisms, and support their application in cardiovascular and environmental health surveillance.

## 1. Introduction

Per- and polyfluoroalkyl substances (PFAS) are a large class of synthetic chemicals widely used over recent decades in industrial and consumer applications, including firefighting foams, solvents, personal care products, textile coatings, non-stick cookware, and food packaging materials [1,2]. Because many of these applications are considered proprietary and not public, it is often difficult to determine which specific PFAS were used and in what amounts [2]. Owing to the strength of their carbon–fluorine bonds, PFAS are highly persistent and resistant to degradation, leading to their accumulation in the environment and in human tissues [1]. Accordingly, measurable concentrations of PFAS are now detected across multiple human biological matrices, reflecting widespread exposure through contaminated food and drinking water, inhalation of indoor air and dust particles, and dermal contact with treated materials [3,4,5]. Among these compounds, long-chain PFAS such as perfluorooctanoic acid (PFOA) and perfluorooctane sulfonate (PFOS) have raised particular concern due to their high-volume production, long biological half-lives in humans, bioaccumulation, and toxicity [6].

A growing body of toxicological and epidemiological evidence has linked PFAS exposure to a broad range of adverse health outcomes, including cardiovascular disease, reproductive and developmental toxicity, adverse pulmonary outcomes including asthma, allergies, infections, and cancer [7,8,9]. Findings from cross-sectional and longitudinal epidemiological and experimental studies increasingly support an association between PFAS exposure and increased cardiovascular risk and mortality, with early vascular alterations emerging as a key mediating link [10,11]. PFAS are thought to promote atherosclerosis through interconnected biological mechanisms, including endothelial dysfunction, oxidative stress, chronic low-grade inflammation, and dysregulation of lipid metabolism, processes that converge to accelerate vascular aging [12].

Consistent with these mechanisms, early manifestations of PFAS-related vascular injury have been documented at both structural and functional levels. Imaging-based studies have reported associations with increased carotid intima–media thickness (CIMT) [13], arterial stiffness [14] and impaired endothelial function [15], both well-established biomarkers of subclinical atherosclerosis. Although direct evidence linking PFAS exposure to coronary artery calcium progression remains limited [16], indirect effects mediated by dyslipidemia and systemic inflammation have been suggested [17,18].

Complementing these observations, molecular studies indicate that PFAS exposure is associated with genetic and epigenetic alterations [19], as well as key pathways involved in vascular damage. These include disruptions in lipid metabolism driven by the activation of peroxisome proliferator-activated receptor alpha, nuclear factor κappa-light-chain-enhancer of activated B cells (NF-κB), and liver X receptor α [20,21], along with increased vascular inflammation [22], reactive oxygen species (ROS) generation [23], and endothelial dysfunction [15], ultimately contributing to vascular injury and atherogenesis.

Taken together, these observations underscore the need for integrated strategies capable of capturing the early, multi-level impact of PFAS on the vascular system. Non-invasive vascular imaging techniques enable the detection of preclinical structural and functional changes, while molecular biomarkers provide insight into early cellular and subcellular perturbations. As these approaches reflect complementary dimensions of vascular damage, their integration offers a promising framework for improving early risk identification in PFAS-exposed populations, although such combined strategies have not yet been systematically evaluated.

In this narrative review, we aim to analyse current evidence on vascular imaging and cellular and molecular biomarkers for the detection of early PFAS-induced vascular aging and to assess their potential integration into cardiovascular risk stratification models. It highlights the importance of longitudinal, population-based studies that integrate multi-omics approaches to validate these tools, elucidate causal relationships, and facilitate their incorporation into cardiovascular risk assessment. Advancing these efforts will be essential to mitigate the long-term burden of PFAS-related vascular disease and to inform effective preventive and regulatory strategies.

## 2. Literature Search

This review was designed as a narrative review aimed at providing a comprehensive overview of the available evidence on the association between PFAS exposure and biomarkers of vascular aging. Although not conducted as a systematic review, a structured literature search with predefined eligibility criteria was adopted to enhance the transparency, reproducibility, and comprehensiveness of study identification and selection. A comprehensive literature search was conducted in PubMed and Scopus to identify studies investigating the association between PFAS exposure and biomarkers of vascular aging. The search was performed on 20 May 2026 and covered two complementary domains: (i) imaging-based biomarkers of vascular aging and (ii) molecular biomarkers implicated in vascular aging, atherosclerosis, and cardiovascular disease. Detailed information about the search strategy is reported in the Supplementary Materials.

No restrictions were applied regarding publication date, language, population characteristics, or study design. Consequently, studies conducted in human populations across all age groups, including paediatric populations, as well as experimental studies in animal models and cell culture systems, were considered eligible. Both cross-sectional and longitudinal studies were included.

Study selection was performed through title and abstract screening followed by full-text assessment of potentially eligible articles. Eligible studies were required to: (i) assess one or more PFAS compounds, including PFAS mixtures; (ii) evaluate at least one imaging or molecular biomarker relevant to vascular aging, atherosclerosis, or cardiovascular disease; and (iii) provide an adequate description of biomarker measurement, acquisition or processing procedures, and statistical methods used to investigate PFAS–biomarker associations. Review articles, editorials, conference abstracts, case reports, and studies lacking relevant biomarker outcomes were excluded. Reference lists of eligible studies and relevant reviews were additionally screened to identify further articles not captured by the electronic database search.

For imaging biomarkers, eligible outcomes included arterial stiffness, typically assessed by pulse wave velocity (PWV), CIMT, endothelial function measured by flow-mediated dilation (FMD), coronary and aortic calcifications assessed by computed tomography (CT) or dual-energy X-ray absorptiometry (DXA), and coronary stenosis evaluated by coronary angiography. Molecular biomarkers included markers of oxidative stress and redox imbalance, inflammation, mitochondrial dysfunction, endothelial dysfunction, DNA damage and genotoxicity, telomere biology, epigenetic regulation (including DNA methylation, microRNAs, and histone modifications), and lipid metabolism.

## 3. Evidence from the Literature

**Figure 1:**
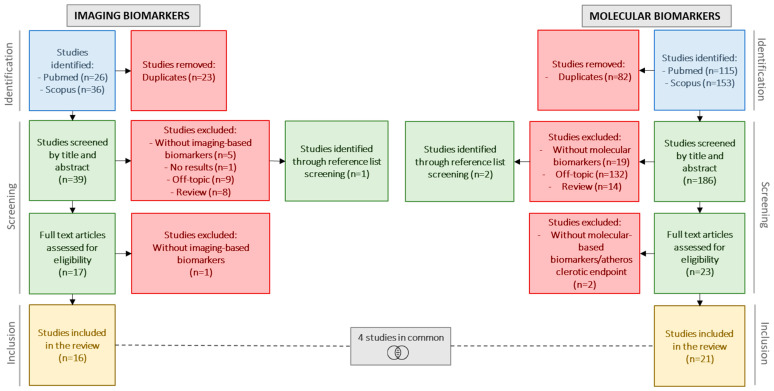
Flowchart illustrating the literature search and study selection process for articles investigating imaging and molecular biomarkers linking PFAS exposure to vascular aging and atherosclerosis.

A total of 62 records were identified through PubMed (26) and Scopus (36) considering imaging biomarkers. Following the removal of 23 duplicates, 39 records underwent title and abstract screening. Of these, 17 articles were assessed for full-text eligibility, resulting in 16 articles included in the final review (Figure 1).

A total of 268 records were identified through PubMed (115) and Scopus (153) considering molecular biomarkers. Following the removal of 82 duplicates, 186 records underwent title and abstract screening. Of these, 23 articles were assessed for full-text eligibility, resulting in 21 articles included in the final review (Figure 1). Four studies overlapped between the two searches.

### 3.1. Imaging Biomarkers

Of the 16 selected studies, 12 are cross-sectional, one is longitudinal [13], one is prospective cohort study with long-term follow-up [16], and the remaining two [24,25] are controlled experimental studies conducted in animal models. Among the 14 studies conducted in humans, two include paediatric cohorts [26,27]. Furthermore, some epidemiological studies are based on data derived from the same population-based research projects. Two studies used data from the Prospective Investigation of the Vasculature in Uppsala Seniors (PIVUS) cohort [13,28], while two others were based on data from the National Health and Nutrition Examination Survey (NHANES) [29,30].

Regarding imaging techniques, 75% of the studies use ultrasound to assess CIMT, PWV (measured at the carotid or abdominal aortic level), or endothelial function through FMD. Dual-energy X-ray absorptiometry (DXA) was used in 12.5% of the studies, while CT and coronary angiography were each used in 6.25% of the studies.

Most studies use standard statistical software and apply a range of regression-based approaches, from conventional linear and logistic models to more advanced methods such as structural equation models, Bayesian kernel machine regression, weighted quantile sum regression, quantile g-computation, restricted cubic splines, mixed-effects models, and principal component analysis (PCA). Analyses are typically adjusted for demographic, socioeconomic, lifestyle, and clinical confounders, and several studies also perform sensitivity analyses and account for multiple testing using methods such as Bonferroni correction, the Holm procedure, or false discovery rate control.

#### 3.1.1. CIMT

Ten studies assessed CIMT by ultrasound; eight of them report a positive association between serum or plasma PFAS concentrations and CIMT. In contrast, the study by Wang et al. [26], conducted on 957 mother–child pairs recruited from six hospitals in Shanghai, reported that higher maternal plasma PFAS concentrations during the first trimester of pregnancy are associated with lower CIMT values in offspring at four years of age. Ultrasound images were analysed using Qlab v10.5 software, yielding a mean CIMT value of 0.41 ± 0.03 mm. Notably, the study did not account for PFAS exposure during the early years of the children’s lives.

In the study by Khalil et al. [31], no association emerged between serum levels of 12 PFAS and CIMT in a cohort of 38 Arizona firefighters aged approximately 50 years with more than five years of service. The authors applied linear models adjusted for age and cardiometabolic risk factors, although no correction for multiple testing was performed. The cohort was compared with a control sample of similar size representing the United States population within the same age range and derived from the 2009–2010 NHANES survey.

In the study by Lind et al. [28], carotid ultrasound images from 1016 Swedish individuals aged 70 years were analysed using the Artery Measurement Software (AMS). A mean CIMT value of 0.88 ± 0.16 mm and a median grayscale median value of 79 ± 23 were obtained. Plaque presence and vascular wall echogenicity were also evaluated. The authors hypothesized a possible role of female sex in the relationship between the eight PFAS detected in blood and atherosclerosis, considered one of the main manifestations of vascular aging.

The paediatric study by Gump et al. [27] included 291 children aged 9–11 years living in Syracuse, New York, and recruited between 2013 and 2017. CIMT was measured both at baseline and during acute psychological stress and was found to be greater in subjects with higher serum perfluorodecanoic acid (PFDA) concentrations. Associations were evaluated using generalized linear models and Bayesian kernel machine regression for PFAS mixture analysis while accounting for potential confounding variables. The authors suggested that PFAS may act as cardiovascular disruptors already during childhood.

In the study by Lin et al. [32], a positive association between PFOS and CIMT was reported in 1425 individuals from two Taiwanese cohorts aged between 12 and 63 years. The two cohorts differed significantly in terms of demographic characteristics. Associations were evaluated through linear regression and structural equation models, adjusted for major confounding variables. CIMT measurements were performed bilaterally, and the average value of both carotid arteries was considered for the analysis.

A positive association between PFOS and CIMT also emerged in the study by Lin et al. [33], in which only four PFAS (PFOA, PFOS, PFDA, and perfluorononanoic acid (PFNA)) exceeded the detection threshold in a cohort of 848 Taiwanese students. The study used data derived from a large-scale urinary screening conducted between 1992 and 2000. CIMT was again measured bilaterally at the common carotid artery. Analyses were performed using logistic regression, with adjustment for confounding variables and correction for multiple testing.

A non-linear relationship between PFOS and CIMT was also reported in the study by Lin et al. [34], which included 664 Taiwanese young adults aged 12–30 years selected from participants in a mass urinary screening conducted in Taipei between 1992 and 2000. Between 2006 and 2008, participants underwent interviews and cardiovascular check-ups. CIMT was measured bilaterally at the common carotid artery over a 10 mm segment. Associations were evaluated through linear and logistic regression models, with adjustment for confounding variables and Bonferroni correction. The association between PFOS and CIMT appeared stronger in women, non-smokers, individuals aged 12–19 years, and subjects with body mass index lower than 24 kg/m^2^.

The longitudinal study by Lind et al. [13] was conducted on a cohort of 1016 Swedish individuals aged 70 years living in the community of Uppsala. During a 10-year follow-up, measurements of eight plasma PFAS and CIMT were performed every five years. Over the observation period, CIMT increased on average by 0.058 ± 0.043 mm, with a greater increase during the first five years; a similar trend was also observed for plasma PFAS concentrations. No association between PFAS and CIMT emerged at baseline, where the median CIMT value was 0.89 ± 0.16 mm. However, longitudinal analysis of repeated measurements within the same subjects revealed a positive association between six PFAS and CIMT. Relationships were evaluated using mixed-effects models and do not appear to be mediated by traditional cardiovascular risk factors. The authors therefore suggested that PFAS may contribute to the atherosclerotic process.

In the study by Liberda et al. [35], a cohort of 535 Indigenous individuals from northern Québec (Canada), aged between 15 and 87 years and originating from nine communities, was analysed. The study was conducted between 2002 and 2009. Serum levels of PFOA, PFOS, and perfluorohexanesulfonic acid (PFHxS) were considered, and carotid ultrasounds were analysed using the Carotid Analyzer for Research software. Since the study included additional pollutants, PCA was applied to reduce data dimensionality, yielding a principal component mainly driven by PFOA, PFOS, and PFHxS. The resulting components were subsequently included in linear regression models. The analysis showed a positive association between PFOA, PFOS, PFHxS, and CIMT. The reported mean CIMT value was 0.673 ± 0.193 mm.

#### 3.1.2. PWV

In the experimental animal study by Wang et al. [25], PWV and CIMT were assessed in ApoE^−/−^ mice divided into four groups: one control group and three groups exposed to increasing equivalent doses of PFOS calculated according to corresponding human exposure levels. Oral administration was performed for 12 weeks, whereas ultrasound measurements are repeated every four weeks and analysed using Vevo 2100 software. The results showed a dose-dependent increase in CIMT and carotid PWV, suggesting a possible pro-atherogenic role of PFOS.

In the controlled experimental study by Lv et al. [24], abdominal aortic ultrasound was performed to assess arterial stiffness through PWV. The study included 28 seven-week-old male C57BL/6J mice divided into four groups: control, 6:2 Cl-PFESA exposure, polystyrene nanoparticle exposure, and combined exposure to both substances. Images were analysed using the Vevo 2010 imaging system, and statistical analysis conducted through ANOVA demonstrated reduced aortic elasticity in exposed groups compared with controls. The results also suggested a possible synergistic effect between the two pollutants on the vascular system.

#### 3.1.3. FMD

The study by Wittkopp et al. [15] is the only one assessing endothelial function through FMD, measured using the brachial artery reactivity test, in a cohort of 94 middle-aged U.S. adults without diagnosed cardiovascular disease and representative of the general population. Ten PFAS were detected above the limit of detection in more than 50% of participants, and the median FMD value was 3.6% (interquartile range, 2.55–5.83). Statistical analyses, including linear regression, weighted quantile sum regression, Bayesian kernel machine regression, and sensitivity analyses, showed an inverse association between PFAS mixtures and FMD. Higher perfluoroheptanoic acid (PFHpA) levels were associated with impaired endothelial function.

#### 3.1.4. Calcifications

Three studies investigating the association between serum PFAS levels and coronary or aortic calcifications were identified. In the study by Osorio-Yáñez et al. [16], conducted on 666 U.S. participants enrolled in the Diabetes Prevention Program, concentrations of six PFAS were measured at baseline and after two years, assigning each participant the average value of the two measurements. CT scans were performed 13–14 years after baseline and allow the assessment of coronary and aortic calcifications, both in the ascending and descending tract, using the Agatston score, which was subsequently categorized for the analysis. Logistic regression models adjusted for major confounding variables, although not for multiple testing, revealed a positive association between plasma PFAS levels and the risk of coronary and aortic calcifications, whereas no association emerged when PFAS mixtures were considered.

The other two studies [29,30], based on cohorts extracted from NHANES 2013–2014, exclusively assess aortic calcifications through dual-energy X-ray absorptiometry. In both cases, calcification scores were derived and subsequently categorized. In the study by Yang et al. [29], involving 1005 middle-aged and older individuals, PFHxS, PFDeA, and PFNA were detected in more than 80% of samples. Analyses conducted using logistic regression, restricted cubic splines, quantile g-computation, and machine learning methods demonstrated a positive association between PFHxS, PFDeA, PFAS mixtures, and calcification risk. Conversely, in the study by Koskela et al. [30], conducted on 913 individuals aged between 40 and 80 years, logistic regression adjusted for covariates and corrected for multiple testing did not reveal significant associations between PFAS and aortic calcifications.

#### 3.1.5. Coronary Stenosis

Finally, the study by Li et al. [36] evaluated the association between PFAS and coronary stenosis, both in terms of disease severity and prognosis, in a cohort of 571 individuals aged between 18 and 80 years diagnosed with coronary stenosis and recruited in 2022 at a hospital in Hebei, China, without known occupational exposure to PFAS. After a one-year follow-up from enrolment, blood sample collection, and coronary angiography, participants underwent interviews and medical record review to assess the occurrence of adverse cardiovascular events. Six PFAS detected in more than 80% of samples were considered in the analysis. Coronary stenosis severity was quantified using the Gensini score and the number of lesioned vessels, both subsequently categorized. Statistical analyses adjusted for major confounding variables and corrected for multiple testing demonstrated a positive association between PFOS and coronary stenosis, both in terms of disease severity and unfavourable prognosis. Table 1 summarizes included studies investigating the associations between PFAS exposure and imaging biomarkers of vascular aging.

### 3.2. Molecular Biomarkers

#### 3.2.1. Oxidative Stress, Inflammation and Endothelial Dysfunction

Nine studies indicate that PFAS exposure may be involved in vascular injury through interconnected mechanisms involving oxidative stress, endothelial dysfunction, and inflammation, ultimately promoting atherosclerotic disease progression.

In vitro studies primarily demonstrated that PFAS may directly induce oxidative stress and inflammatory activation in endothelial cells. In human umbilical vein endothelial cells (HUVECs), Liao et al. [37] showed that PFOS exposure induced a dose- and time-dependent increase in intracellular ROS production, accompanied by upregulation of pro-inflammatory mediators, including interleukin 1β (IL-1β), IL-6, cyclooxygenase-2 (COX-2), intercellular adhesion molecule-1 (ICAM-1), and P-selectin. PFOS also enhanced THP-1 monocyte adhesion to endothelial cells, a key early event in atherogenesis, indicating that oxidative stress may promote endothelial activation and vascular inflammation.

Additional mechanistic evidence was provided by Cui et al. [38], who demonstrated that PFOS exposure may contribute to ferroptosis-related endothelial injury in HUVECs. Specifically, PFOS increased lipid ROS accumulation and expression of acyl-CoA synthetase long-chain family member 4, while reducing glutathione peroxidase 4, ferritin heavy chain 1, heme oxygenase-1, and nitric oxide levels, supporting activation of ferroptotic pathways and oxidative endothelial damage.

More recent transcriptomic analyses further clarified the molecular pathways linking PFAS exposure to endothelial inflammation. Vajeethaveesin et al. [39] demonstrated that PFOS activates the heme-regulated inhibitor/eukaryotic initiation factor 2α/activating transcription factor 4 branch of endoplasmic reticulum stress (ERS) in endothelial cells, resulting in NF-κB- and JAK2/STAT3-mediated upregulation of COX-2, ICAM-1, and IL-6.

Similarly, Zhang et al. [40] showed that exposure to sodium p-perfluorous nonenoxybenzene sulfonate, an emerging PFOS substitute, may contribute to ROS generation and activated the PERK–eIF2α–ATF4 branch of ERS in HUVECs. Sequential inhibition experiments revealed that ROS accumulation occurs upstream of ERS activation, which subsequently triggered NF-κB signaling, inflammatory responses, monocyte adhesion, impaired endothelial migration, and endothelial barrier dysfunction.

Using a prenatal rat exposure model, Dangudubiyyam et al. [41] demonstrated that maternal PFOS exposure is related to persistent hypertension and impaired vascular relaxation in adult offspring, indicating long-term endothelial dysfunction following developmental exposure. Endothelium-dependent vasodilation was significantly reduced in both sexes, while females additionally exhibited impaired endothelium-independent relaxation. Mechanistically, PFOS exposure decreased endothelial nitric oxide synthase (eNOS) activity in males and reduced both eNOS expression and activation in females, suggesting sustained disruption of NO signaling and vascular homeostasis.

In ApoE^−/−^ mice, vascular alterations induced by PFOS were accompanied by macrophage polarization toward the pro-inflammatory M1 phenotype, characterized by increased inducible nitric oxide synthase, tumor necrosis factor alpha, IL-6, and IL-1β expression together with suppression of anti-inflammatory M2 markers, including cluster of differentiation 206, arginase-1, and IL-10. The inflammatory effects were mediated by activation of NF-κB signaling pathway, and pharmacological inhibition of NF-κB significantly attenuated PFOS-related inflammatory responses [25].

Lv et al. [24] demonstrated that co-exposure to the PFOS substitute F-53B and polystyrene nanoplastics is associated with endothelial dysfunction and vascular remodeling through the activation of NF-κB/NLR family pyrin domain containing 3 (NLRP3) inflammasome pathways. Increased expression of NLRP3, apoptosis-associated speck-like protein containing a CARD, cleaved caspase-1 (CASP1), gasdermin D, and IL-1β was associated with endothelial pyroptosis, whereas NF-κB inhibition markedly attenuated inflammasome activation and endothelial cell death. Similarly, Zhang et al. [40] confirmed in ApoE^−/−^ mice that OBS exposure may contribute to endothelial injury, oxidative stress, collagen deposition, and vascular inflammation. Overall, animal studies consistently demonstrate that PFAS exposure is linked to vascular remodeling and atherosclerosis through persistent endothelial dysfunction, oxidative stress, and inflammatory activation.

Human studies supported the clinical relevance of these experimental findings by linking PFAS exposure to biomarkers of endothelial injury and cardiovascular risk. In a cohort of 848 adolescents and young adults, Lin et al. [33] reported positive associations between serum PFOS concentrations and circulating endothelial and platelet microparticles, including CD31+/CD42a− and CD31+/CD42a+, biomarkers of endothelial and platelet apoptosis. These findings suggested that PFOS-associated endothelial injury may contribute to early vascular remodeling and subclinical atherosclerosis. Further epidemiological evidence was provided by Zhao et al. [42], who reported associations between PFAS mixture exposure and hypercholesterolemia, elevated LDL cholesterol, hypertension, and hyperuricemia. The analyses demonstrated that inflammatory biomarkers, particularly high-sensitivity C-reactive protein and serum ferritin, explain a substantial proportion of these associations, in some cases accounting for more than 80% of the observed effect. These findings support chronic low-grade inflammation as a major biological pathway, linking PFAS exposure to cardiometabolic and vascular disease in human populations.

#### 3.2.2. Lipid Metabolism Dysregulation

Experimental studies provide mechanistic evidence supporting PFAS-related disruption of lipid metabolism. Specifically, Connolly et al. [43] investigated the effects of PFOS and PFOA in human U937-derived macrophages and demonstrated a marked increase in intracellular lipid and cholesterol accumulation, a hallmark of foam cell formation involved in early atherosclerotic plaque development. Both PFOS and PFOA activate PPAR signaling, and pharmacological inhibition of this receptor partially reversed PFAS-associated lipid accumulation, indicating a direct role for PPARγ signaling in macrophage lipid dysregulation. This group further showed that PFOS and PFOA alter the expression of multiple genes involved in lipid metabolism and inflammation. PFAS exposure increased IL-1β, COX-2, AKR1C3, plasminogen activator inhibitor-2, and matrix metalloproteinase (MMP) 1 and MMP-12, while reducing CYP8B1 and LSS, both involved in cholesterol and bile acid metabolism. These molecular changes were accompanied by activation of nuclear factor erythroid 2-related factor 2 signaling and oxidative stress responses, suggesting that PFAS-associated lipid accumulation occurs within a broader inflammatory and redox imbalance that promotes plaque formation and progression [43]. Further mechanistic support was provided by Chai et al., who identified key molecular targets linking PFAS exposure to lipid metabolism and atherosclerosis, including albumin, carbonic anhydrase 2 (CA2), PPARγ, AKT1, STAT3, MMP-9, CASP1, CASP3, and IL-10, reinforcing a network-level connection between PFAS exposure and cardiovascular disease [44].

In vivo evidence consistently shows that PFAS exposure disrupts systemic lipid metabolism and accelerates atherogenesis. Roth et al. investigated hyperlipidemic LDLr^−/−^ mice fed an atherogenic diet and demonstrated that exposure to a mixture of five PFAS (PFOA, PFOS, PFNA, PFHxS, and GenX) increases circulating cholesterol concentrations. Total cholesterol increased modestly by approximately 10%, but lipoprotein distribution shifted more dramatically, including a 25% increase in intermediate-density lipoproteins and a 206% increase in the highly atherogenic LDL7 subfraction.

Roth et al. further showed that transcriptomic profiling of isolated aortic macrophages reveals extensive metabolic reprogramming, with more than 900 genes differentially expressed following PFAS exposure. Upregulated pathways were enriched for lipid metabolism, fatty acid synthesis, cholesterol handling, and foam cell development. Strongly induced genes included fatty acid-binding protein 4 and fatty acid synthase, together with inflammatory chemokines such as C-X-C motif chemokine ligand (CXCL) 2 (CXCL2) and CXCL17. These changes collectively resemble a foam-cell macrophage phenotype, indicating that PFAS exposure may promote both systemic dyslipidemia and vascular lipid accumulation during early atherogenesis [45].

Zhang et al. further demonstrated that in ApoE^−/−^ mice, chronic exposure to PFOS or the replacement compound OBS accelerates dyslipidemia and atherosclerosis. Although PFOS showed greater tissue accumulation, OBS may be associated with faster increases in circulating lipid abnormalities and stronger vascular inflammatory responses. Mechanistically, OBS activated NF-κB and MAPK/ERK signaling pathways and increases endothelial permeability, suggesting that replacement PFAS compounds retain or even enhance vascular toxicity [46]. Moreover, Du et al. performed integrative toxicogenomic analyses and showed that lipid metabolism and atherosclerosis are among the most consistently enriched biological pathways following mixed PFAS exposure. Transcriptomic profiling further revealed enrichment of lipid handling, inflammatory signaling, and apoptosis, and JAK-STAT pathways, indicating that PFAS may contribute to metabolic disruption occurring within a broader network of immune and cardiovascular regulatory alterations [47].

In a recent analysis of NHANES data from 2005–2018, Pan et al. demonstrated that serum concentrations of PFOS, PFOA, and PFNA are positively associated with LDL-C, total cholesterol, triglycerides, and the atherogenic index of plasma in adolescents. They further showed that mixture analyses confirm a cumulative effect of PFAS exposure on lipid abnormalities, supporting the hypothesis that PFAS may be involved in early metabolic alterations preceding overt cardiovascular disease. Interestingly, red blood cell folate partially attenuated several of these associations, particularly those involving triglycerides and atherogenic index of plasma, suggesting that one-carbon metabolism modulates PFAS-related lipid disturbances.

Network toxicology analyses in the same study identified several molecular targets involved in lipid regulation and atherosclerosis, including PPARγ, IL-10, CASP1, CA2, and albumin, further supporting a mechanistic link between PFAS exposure and cardiovascular risk [48].

#### 3.2.3. Epigenetic Modulation

Lin et al. [32] provided some of the first epidemiological evidence supporting a role for DNA methylation in PFAS-associated vascular injury. In a cross-sectional study including 1425 young and middle-aged Taiwanese individuals, serum PFOS concentrations were positively associated with global DNA methylation levels, assessed using the 5-methyl-2′-deoxycytidine/deoxyguanosine (5mdC/dG) ratio. Structural equation modelling suggested that PFOS exposure may influence vascular aging both directly and indirectly through alterations in DNA methylation, highlighting a potential mediating role of epigenetic regulation in PFAS-related vascular effects.

Liu et al. [49] expanded these observations through a longitudinal epigenome-wide association study investigating prenatal PFAS exposure and DNA methylation at birth and during adolescence. Among mother–child pairs from the HOME cohort, the authors identified 435 cytosine-phosphate-guanine dinucleotide (CpG) sites significantly associated with gestational PFAS exposure, including 413 CpGs associated with PFNA, twelve with PFOA, eight with PFHxS and two with PFOS. Importantly, many of these methylation signatures were detectable both in cord blood and in peripheral blood collected at 12 years of age, suggesting long-term persistence of PFAS-associated epigenetic modifications. Several affected CpGs mapped to genes involved in cardiovascular disease, metabolic regulation, cognitive function and kidney disease, while pathway enrichment analyses indicated alterations in biological processes related to cellular development and disease susceptibility. These findings support the hypothesis that prenatal PFAS exposure may induce durable epigenetic programming effects with potential long-term cardiometabolic consequences.

In addition to DNA methylation, increasing evidence indicates that PFAS may influence cardiovascular risk through dysregulation of miRNAs, key post-transcriptional regulators of gene expression. Xu et al. [50] investigated women from the highly PFAS-exposed Ronneby cohort in Sweden, where drinking water contamination had resulted in exceptionally elevated serum PFOS and PFHxS concentrations. Using next-generation sequencing followed by qPCR validation, the authors identified significant downregulation of miR-101-3p, miR-144-3p, and miR-19a-3p with increasing PFAS exposure. Functional enrichment analyses linked these miRNAs to cardiovascular disease, vascular function, and inflammatory pathways. Furthermore, predicted target genes include PPARα, DNA methyltransferase 3 alpha (DNMT3A), 3-hydroxy-3-methylglutaryl-CoA reductase, prostaglandin-endoperoxide synthase 2, and epidermal growth factor receptor, suggesting potential interactions between PFAS exposure, epigenetic regulation, lipid metabolism, and inflammatory signaling. Particularly noteworthy is the identification of DNMT3A, a key DNA methyltransferase, which provides a mechanistic connection between PFAS-associated miRNA dysregulation and DNA methylation pathways.

More recently, Li et al. [51] examined associations between PFAS exposure and circulating miRNA profiles in two independent cohorts from the United States and Greece. PFAS concentrations were associated with altered expression of hundreds of circulating miRNAs, with miR-148b-3p and miR-29a-3p emerging as the most consistently downregulated transcripts across both cohorts. Pathway analyses demonstrated enrichment of cardiovascular disease, inflammatory, and carcinogenesis-related pathways among PFAS-associated miRNAs. Notably, members of the miR-29 family have previously been implicated in extracellular matrix remodeling, fibrosis, and vascular aging, suggesting that PFAS-related miRNA alterations may contribute to long-term vascular dysfunction through modulation of tissue remodeling and inflammatory processes.

Karakuş et al. [52] employed network toxicology and bioinformatic approaches to identify molecular pathways linking PFAS exposure and cardiovascular disease. Their analyses identified several PFAS-associated microRNAs, including miR-130b-3p, miR-130a-3p, and miR-129-5p, as potential mediators of cardiovascular toxicity. Functional enrichment analyses revealed significant involvement of pathways related to lipid metabolism, fatty acid processing, and cardiovascular regulation, reinforcing the concept that epigenetic mechanisms may integrate PFAS exposure with downstream cardiometabolic dysfunction.

Consistent with these findings, Zhang et al. [53] reported that exposure to the PFOS-substitute OBS is associated with alterations in endothelial miRNA-mRNA regulatory networks, identifying several cardiovascular disease-associated targets and further supporting the role of post-transcriptional epigenetic mechanisms in PFAS-related vascular toxicity. Table 2 summarizes studies investigating the associations between PFAS exposure and molecular biomarkers of vascular aging.

## 4. Critical Appraisal of the Evidence

This narrative review synthesizes current evidence on the relationship between PFAS exposure and early vascular aging, integrating findings from both imaging-based and molecular biomarkers. Overall, the available literature suggests a possible association between PFAS exposure and subclinical vascular damage, likely mediated by complex and interconnected biological mechanisms. A total of 37 original studies were included in our analysis, of which 4 were common to both the imaging-based and molecular biomarker categories.

From an imaging perspective, the strongest evidence concerns CIMT, which was evaluated in 10 of the 16 included studies. Most studies reported a positive association between circulating PFAS concentrations and increased CIMT. These findings were observed across heterogeneous populations, including both adults and children, suggesting that PFAS-related vascular alterations may begin early in life. Nevertheless, some inconsistencies remain. A limited number of studies reported null or inverse associations, potentially reflecting differences in study design, timing of exposure assessment (e.g., prenatal versus postnatal), sample size, or residual confounding. In particular, the only two studies evaluating CIMT in children [26,27] yielded apparently discordant findings. Wang et al. assessed maternal PFAS concentrations only during the first trimester of pregnancy and did not account for maternal exposure during the subsequent trimesters or postnatal exposure of the offspring during the first four years of life, which may have resulted in an incomplete characterization of PFAS exposure and potential exposure misclassification [26]. In contrast, Gump et al. directly assessed PFAS exposure in children aged 9–11 years [27]. Additionally, the two studies evaluated vascular outcomes at markedly different stages of vascular development, making direct comparison difficult. Differences in the developmental stage of the vascular system at the time of outcome assessment may also have contributed to the observed discrepancies, although the available evidence is insufficient to determine whether PFAS exert age-dependent vascular effects. Moreover, the inverse association reported by Wang et al. contrasts with the overall body of experimental and epidemiological evidence suggesting detrimental vascular effects of PFAS exposure. Whether this finding reflects developmental differences, methodological factors, residual confounding, or exposure misclassification remains uncertain and warrants further investigation. Overall, these methodological and biological differences may partly explain the apparent discordant findings and suggest that the results should be interpreted with caution. Notably, the longitudinal study by Lind et al. demonstrated that associations became evident when repeated measurements were analysed, underscoring the importance of considering temporal dynamics and cumulative exposure in the assessment of PFAS-related vascular effects [13]. Evidence for other imaging biomarkers, such as PWV, FMD, and vascular calcifications, is more limited but tendentially consistent with a detrimental vascular effect. Experimental animal studies provide robust support for increased arterial stiffness following PFAS exposure [24,25], while the single human study assessing FMD suggests impaired endothelial function in relation to PFAS mixtures [15]. Findings on calcifications and coronary stenosis are suggestive but heterogeneous, and further large-scale longitudinal studies are needed to clarify their role in PFAS-related cardiovascular risk. In the study by Osorio-Yáñez [16], PFAS exposure was assessed at baseline and after two years, whereas vascular calcification was evaluated approximately 13–14 years later. Therefore, the observed associations should be interpreted with caution, as PFAS exposure was characterized only during the early phase of follow-up and was not reassessed before the CT examination. Changes in PFAS exposure over the subsequent years may have resulted in some degree of long-term exposure misclassification, potentially attenuating or influencing the observed associations.

From a molecular perspective, a substantial body of experimental and epidemiological evidence supports multiple converging pathways linking PFAS exposure to vascular injury. Oxidative stress, inflammation, and endothelial dysfunction emerge as central mechanisms. In vitro and in vivo studies consistently demonstrate activation of pro-inflammatory signalling pathways and increased ROS production. These alterations may promote endothelial activation and vascular remodelling, both of which are key processes in early atherogenesis. In parallel, dysregulation of lipid metabolism represents another major pathway. PFAS exposure has been shown to alter lipid metabolism both at the cellular level, promoting foam cell formation, and at the systemic level, inducing dyslipidaemia and atherogenic lipoprotein profiles. These effects are largely mediated through nuclear receptor signalling and are supported by both experimental models and human epidemiological data, including large population-based studies. Emerging evidence also highlights the role of epigenetic mechanisms in mediating PFAS-induced vascular effects. DNA methylation and microRNA dysregulation appear to link environmental exposure to long-term changes in gene expression relevant to vascular function, inflammation, and lipid metabolism. Notably, some epigenetic alterations are detectable from birth and persist into adolescence, supporting the hypothesis of early-life programming effects. Although promising, these findings remain largely exploratory and require validation in longitudinal and multi-omics frameworks.

To date, only four studies have integrated imaging and molecular biomarkers to assess PFAS-related vascular injury, with findings suggesting that PFAS exposure may be associated with both structural vascular alterations and underlying molecular mechanisms of disease. Specifically, experimental evidence indicates that PFOS and related compounds induce arterial wall thickening, reduced vascular elasticity, endothelial dysfunction, and inflammatory activation. In animal models, these cellular changes are accompanied by increased arterial stiffness, IMT, and atherosclerotic plaque burden. Human studies further support these findings, linking higher PFOS exposure to endothelial injury, increased circulating endothelial and platelet-derived microparticles, elevated CIMT, and alterations in DNA methylation. Collectively, these studies suggest mechanistic links between upstream molecular perturbations with downstream structural vascular changes.

From a conceptual perspective, molecular and imaging biomarkers represent complementary and sequential stages of PFAS-induced vascular injury. Molecular markers capture early and potentially reversible biological responses to exposure, whereas imaging biomarkers reflect downstream structural and functional vascular changes. Their integration may therefore improve the identification of individuals across the full spectrum of disease progression, from molecular perturbations to established vascular remodelling. Following exposure, PFAS accumulation in vascular and metabolically active tissues may trigger oxidative stress, mitochondrial dysfunction, inflammatory pathway activation, endothelial injury, and lipid metabolism disruption. These early events promote endothelial dysfunction, increased vascular permeability, leukocyte adhesion, smooth muscle cell proliferation, and extracellular matrix remodelling. Epigenetic modifications, including DNA methylation and microRNA dysregulation, may further contribute to the persistence of these alterations over time. Collectively, these mechanisms are consistent with the development of vascular abnormalities detectable by imaging, including increased CIMT, arterial stiffness, impaired endothelial function, and vascular calcification.

Although direct longitudinal evidence remains limited, this sequence supports a model in which persistent molecular dysregulation precedes and potentially predicts subsequent imaging changes such as CIMT and PWV. Prospective studies integrating repeated molecular profiling with serial vascular imaging are therefore needed to validate this temporal relationship and to evaluate the predictive value of combined biomarker approaches.

This concept may also be translated into a multimodal cardiovascular risk stratification framework. In clinical and public health settings, molecular biomarkers could serve as indicators of PFAS-related biological effects, while vascular imaging may help refine risk assessment in individuals with evidence of ongoing vascular involvement. Such a sequential approach aligns with established multimodal risk prediction strategies in cardiovascular medicine, where combining biochemical and imaging markers improves risk classification beyond traditional factors. Although this approach has not yet been validated specifically in PFAS-exposed populations, existing evidence supports its potential applicability and warrants future investigation.

Despite these advances, several limitations should be considered. The current literature is largely based on cross-sectional studies, limiting the assessment of temporal relationships and causal inference. In addition, PFAS exposure is frequently assessed at a single time point, which may not accurately capture long-term exposure patterns or critical windows of susceptibility. Considerable methodological heterogeneity further limits direct comparability across studies. Differences include the number and type of PFAS congeners evaluated (ranging from a few compounds to broad multi-analyte panels), the biological matrices used for exposure assessment, the timing of exposure evaluation (prenatal, childhood, or adulthood), the characteristics of the study populations, and the vascular imaging protocols and outcome definitions adopted. These variations likely contribute to inconsistencies in the reported findings and hinder meaningful quantitative comparisons, making it difficult to distinguish true biological variability from differences attributable to study design and measurement approaches.

An additional challenge arises from the complexity of real-world exposure, which typically involves mixtures of PFAS and other environmental contaminants, whereas many epidemiological studies have historically examined individual compounds, particularly PFOS and PFOA. More recent investigations increasingly employ mixture-oriented analytical approaches, including Bayesian Kernel Machine Regression (BKMR), Weighted Quantile Sum (WQS) regression, Quantile G-Computation (QGC), and Principal Component Analysis (PCA), to better reflect environmental co-exposures. The adoption of these methods has increased substantially in recent years, reflecting the recognition that human exposure occurs as complex mixtures rather than as isolated compounds. However, these statistical approaches address different scientific questions. BKMR is particularly useful for identifying non-linear exposure–response relationships and interactions among PFAS congeners. WQS regression estimates the overall effect of the mixture while identifying compounds that contribute most strongly to the observed association. Quantile G-Computation allows both positive and negative contributions of individual chemicals within the same mixture, whereas PCA primarily reduces exposure dimensionality by summarizing correlated PFAS into latent components. Although these complementary approaches provide different perspectives on mixture effects, their findings should not be interpreted as evidence of a uniform PFAS class effect. Nevertheless, they consistently suggest that legacy long-chain PFAS—particularly PFOS and, to a lesser extent, PFOA—are the principal contributors to the observed vascular associations, whereas evidence for shorter-chain and replacement PFAS remains limited and inconsistent. Methodological differences among these analytical frameworks, however, preclude direct comparison of their results and highlight the need for greater standardization of mixture analyses in future epidemiological studies.

Although these methods provide complementary insights, their findings should not be interpreted as evidence of a uniform PFAS class effect. Across both single-compound and mixture-based analyses, the most consistent associations with vascular biomarkers are observed for legacy long-chain PFAS, especially PFOS and, to a lesser extent, PFOA, whereas evidence for shorter-chain and replacement PFAS remains limited and inconsistent. These differences are biologically plausible and may reflect the distinct physicochemical and toxicokinetic characteristics of individual PFAS congeners. Long-chain PFAS exhibit greater bioaccumulation, longer biological half-lives, stronger protein-binding affinity, and more pronounced interactions with molecular pathways involved in lipid metabolism, oxidative stress, and inflammation than shorter-chain alternatives. In addition, sulfonated PFAS such as PFOS may exert stronger vascular effects than carboxylated compounds because of differences in tissue distribution and cellular behavior. Nevertheless, current evidence remains insufficient to establish congener-specific vascular toxicity, underscoring the need for studies specifically designed to evaluate structure–activity relationships.

Another methodological limitation is the inconsistent application of multiple-testing corrections across epidemiological studies. Many investigations simultaneously evaluate numerous PFAS congeners against multiple vascular outcomes, yet only a subset applies correction procedures such as Bonferroni adjustment, Holm correction, or false discovery rate control. Consequently, statistically significant associations reported without correction should be interpreted cautiously because of the increased likelihood of type I error. Conversely, studies applying more stringent correction procedures may report fewer significant associations, reflecting greater statistical robustness rather than weaker biological effects. Similarly, null findings following multiple-testing correction should not necessarily be interpreted as evidence for the absence of an association but rather as the consequence of more rigorous control of false-positive results. Greater methodological harmonization, including consistent approaches to multiple-comparison adjustment, would substantially improve the comparability, reproducibility, and overall reliability of future PFAS epidemiological studies.

Overall, these methodological limitations temper the strength of the conclusions, as several studies are affected by small sample sizes, heterogeneous designs, and variability in exposure assessment, outcome definitions, and study design. These sources of heterogeneity limit cross-study comparability and reduce the strength of the overall evidence of synthesis. Nevertheless, the available evidence supports a biologically plausible association between PFAS exposure and early vascular aging.

Further research is needed to strengthen causal inference, identify susceptible populations, and validate clinically relevant biomarkers. Future studies should prioritize large-scale longitudinal and multicenter designs integrating advanced vascular imaging, human biomonitoring, and multi-omics approaches—including epigenomics, transcriptomics, proteomics, metabolomics, and lipidomics. Such integrated strategies may improve our understanding of the biological mechanisms underlying PFAS-related vascular damage and facilitate the development of sensitive biomarkers for early detection and cardiovascular risk prediction in exposed populations.

## 5. Conclusions and Future Perspectives

Current evidence supports PFAS exposure as an emerging environmental determinant of early vascular aging. Although the available evidence remains heterogeneous and largely observational, imaging and molecular studies have identified promising indicators of subclinical vascular injury that may improve the understanding of PFAS-related cardiovascular risk. Rather than establishing definitive clinical biomarkers, current findings suggest that the integration of structural, functional, and molecular approaches may provide a more comprehensive assessment of early vascular alterations associated with PFAS exposure.

Future research should prioritize large longitudinal studies with standardized PFAS exposure assessment, repeated vascular phenotyping, and improved characterization of real-world PFAS mixtures. Attention should focus on identifying sensitive biomarkers across different life stages and validating multimodal approaches that integrate imaging, molecular, and multi-omics data. The application of machine learning and other data-driven approaches may further contribute to identifying exposure patterns, high-risk subgroups, and biomarker signatures capable of improving cardiovascular risk prediction beyond traditional approaches. However, these strategies require robust validation, harmonization of methodologies, and demonstration of clinical utility before translation into routine practice.

From a clinical perspective, validated imaging and molecular biomarkers could support earlier identification of individuals at increased cardiovascular risk and contribute to personalized prevention strategies in populations exposed to PFAS. Such approaches may be particularly relevant for populations with documented higher PFAS exposure, including occupationally exposed workers, residents of PFAS-contaminated communities, and potentially other vulnerable groups with sustained environmental exposure.

From a public health perspective, biomarker-based surveillance may complement environmental monitoring by improving risk assessment and informing strategies aimed at reducing PFAS exposure. At present, however, no PFAS-specific biomarker cut-off values have been validated for cardiovascular risk stratification, underscoring the need for prospective studies to establish clinically meaningful thresholds before routine implementation.

Overall, advancing an integrated precision environmental health approach will require collaboration across cardiovascular research, environmental epidemiology, molecular biology, and data science to translate mechanistic insights into effective prevention and risk reduction strategies.

## Figures and Tables

**Table 1:** Summary of studies investigating the associations between PFAS exposure and imaging biomarkers of vascular aging, including carotid intima–media thickness, pulse wave velocity, endothelial function, coronary and aortic calcifications, and coronary stenosis.

Main Findings	Effect Modifiers	Statistical Analysis	Imaging Biomarker	PFAS	Population	Study Type	First Author and Year
Certain PFAS positively associated with CIMT and GSM-CIMT; possible influence of female sex	Sex, BMI, smoking status, BP, exercise habits, energy and alcohol intake, LDL-C, HDL-C, triglycerides, diabetes, educational level	Linear and logistic regression, structural equation models	CIMT	PFHpA PFHxS L-PFOS PFOA PFNA PFDA PFOSA PFUnDA	1016 Swedish 70-year-olds	Cross-sectional	Lind et al., 2017 [28]
Maternal PFAS concentrations inversely associated with offspring CIMT	Maternal age, pre-pregnancy BMI, household income, educational level, offspring sex	Linear regression, BKMR	CIMT	PFOA PFOS PFNA PFDA PFUA PFHxS PFDoA PFBS PFHpA PFOSA	957 mother–child pairs recruited from six hospitals in Shanghai	Prospective	Wang J et al., 2023 [26]
PFDA positively associated with CIMT	Age, sex, BMI, ethnicity, blood mercury levels, urinary total arsenic, parental household income, occupation, educational level	General linear modelling, BKMR	CIMT	PFOS PFOA PFNA PFHxS PFDA	291 children aged 9–11 years from Syracuse, New York	Cross-sectional	Gump et al., 2023 [27]
PFOS positively correlated with mean CIMT	Age, sex, BMI, smoking status, alcohol consumption, household income, hypertension, diabetes, hyperlipidaemia	Linear regression, structural equation models	CIMT	PFOS	1425 Taiwanese individuals aged 12–63 years from two cohorts	Cross-sectional	Lin et al., 2022 [32]
Positive association between PFOS and CIMT	Age, sex, BMI, smoking status, systolic BP, LDL-C, HDL-C, triglycerides, HOMA-IR, hs-CRP	Logistic regression, Bonferroni correction	CIMT	PFOA PFOS PFNA PFUA	848 Taiwanese students aged 12–30 years	Cross-sectional	Lin et al., 2016 [33]
PFOS levels positively associated with mean CIMT	Age, sex, BMI, smoking status, systolic BP, LDL-C, triglycerides, hs-CRP, HOMA-IR	Linear regression, logistic regression, Bonferroni correction	CIMT	PFOA PFOS PFNA PFUA	664 Taiwanese individuals aged 12–30 years from the Taipei area	Cross-sectional	Lin et al., 2013 [34]
Positive association between six PFAS and CIMT over 10 years	Sex, BMI, smoking status, systolic BP, baseline PFAS levels, HDL-C, LDL-C, fasting glucose, triglycerides, statin use	Mixed-effects models, Bonferroni correction	CIMT	PFHpA PFHxS PFOS PFOA PFNA PFDA PFOSA PFUnDA	1016 Swedish 70-year-olds at baseline	Longitudinal	Lind et al., 2018 [13]
No association between PFAS and CIMT	Age, smoking status, exercise habits, family history of heart disease, HDL-C, triglycerides, fasting glucose, sICAM-1, hs-IL-6	Linear models	CIMT	PFBuS PFDeA PFDoA PFHpA PFHxS PFNA PFOSA Et-PFOSA-AcOH Me-PFOSA-AcOH PFOS PFOA PFUA	38 male firefighters from Arizona aged 49–54 years with >5 years of service; 49 controls aged 49–54 years from the 2009–2010 NHANES study	Cross-sectional	Khalil et al., 2020 [31]
PFOA, PFOS, and PFHxS positively associated with CIMT	Age, sex, BMI, smoking status, systolic BP, LDL-C, Apo-B, triglycerides, TNF-α, hs-CRP, ox-LDL	PCA, linear regression, sensitivity analysis, Holm method	CIMT	PFOA PFOS PFHxS	535 Indigenous individuals from nine communities in northern Quebec, aged 15–87 years	Cross-sectional	Liberda et al., 2019 [35]
PFOS exposure associated with increased CIMT, arterial stiffness, aortic plaque burden, and plaque vulnerability	ns	ANOVA, LSD test, Dunnett’s test	CIMT, left common carotid artery PWV	PFOS	Male ApoE^−/−^ mice aged 7 weeks at baseline	Controlled experimental	Wang D et al., 2023 [25]
Exposure associated with increased abdominal aortic PWV	ns	One-way ANOVA, LSD test, Dunnett’s test, factorial ANOVA	Abdominal aortic PWV	6:2 Cl-PFESA (trade name F-53B)	28 adult male C57BL/6J mice	Controlled experimental	Lv et al., 2025 [24]
PFHpA inversely associated with FMD	Age, sex, BMI, smoking status, ethnicity, HbA1c, hypertension, hypercholesterolemia, waist circumference, systolic BP, diastolic BP	Linear regression, WQS, BKMR, sensitivity analysis	FMD	PFPeA PFHxA PFHpA PFOA PFNA PFDA PFUnDA PFBS PFHxS PFOS	94 adults with a mean age of 55 years from the United States without known cardiovascular disease	Cross-sectional	Wittkopp et al., 2022 [15]
Positive association between PFOS, n-PFOS, and Et-FOSAA and coronary/aortic calcifications over 13–14 years	Age, sex, BMI, ethnicity, educational level, smoking status, treatment assignment, statin use	Logistic regression, sensitivity analysis	Coronary and thoracic aortic calcifications	PFOS PFOA PFHxS EtFOSAA MeFOSA-A PFNA	666 prediabetic adults from 27 centres in the United States, enrolled in the Diabetes Prevention Program	Prospective cohort study with long-term follow-up	Osorio-Yanez et al., 2021 [16]
PFHxS, PFDeA, and PFAS mixtures associated with increased calcification risk	Age, sex, BMI, ethnicity, smoking status, education, household income, phosphorus, vitamin B12, TC, total calcium, AST, ALT, HbA1c, total 25-hydroxyvitamin D, uric acid, eGFR, hypertension, diabetes	Logistic regression, sensitivity analysis, RCS, QGC, XGBoost with SHAP, mediation analysis	Abdominal aortic calcifications	PFDeA PFHxS PFNA	1005 middle-aged and older adults from the United States enrolled in the 2013–2014 NHANES study	Cross-sectional	Yang et al., 2025 [29]
No significant association between PFAS and abdominal aortic calcifications	Age, sex, ethnicity, cotinine, household income	Logistic regression, Bonferroni correction	Abdominal aortic calcifications	PFOA PFOS PFHxS PFNA	913 subjects aged 40–80 years enrolled in the 2013–2014 NHANES study	Cross-sectional	Koskela et al., 2022 [30]
Positive association between PFOS and coronary stenosis	Age, sex, BMI, smoking status, drinking, educational level	Logistic regression, Cox regression, RCS, threshold effect model, BKMR, QGC, sensitivity analysis, FDR	Coronary stenosis	PFOA PFOS PFNA PFHxS PFUnDA PFDA	571 subjects with acute coronary syndrome aged 18–80 years from Hebei, China	Cross-sectional	Li et al., 2024 [36]

Abbreviations: 6:2 Cl-PFESA (F-53B): 6:2 chlorinated polyfluoroalkyl ether sulfonic acid; ANOVA: analysis of variance; Apo-B: apolipoprotein B; AST: aspartate aminotransferase; BKMR: Bayesian kernel machine regression; BMI: body mass index; BP: blood pressure; CIMT: carotid intima–media thickness; eGFR: estimated glomerular filtration rate; EtFOSAA: N-ethyl perfluorooctane sulfonamidoacetic acid; Et-PFOSA-AcOH: N-ethyl perfluorooctane sulfonamidoacetic acid; FDR: false discovery rate; FMD: flow-mediated dilation; GSM-CIMT: grayscale median carotid intima–media thickness; HbA1c: glycated haemoglobin A1c; HDL-C: high-density lipoprotein cholesterol; HOMA-IR: homeostasis model assessment of insulin resistance; hs-CRP: high-sensitivity C-reactive protein; hs-IL-6: high-sensitivity interleukin-6; ICAM-1: Intercellular adhesion molecule-1; LDL-C: low-density lipoprotein cholesterol; LSD: least significant difference; LT: alanine aminotransferase; L-PFOS: linear perfluorooctane sulfonate; MeFOSAA: N-methyl perfluorooctane sulfonamidoacetic acid; Me-PFOSA-AcOH: N-methyl perfluorooctane sulfonamidoacetic acid; NHANES: National Health and Nutrition Examination Survey; ns: not specified; ox-LDL: oxidized low-density lipoprotein; PCA: principal component analysis; PFAS: per- and polyfluoroalkyl substances; PFBS: perfluorobutane sulfonic acid; PFBuS: perfluorobutane sulfonate; PFDA: perfluorodecanoic acid; PFDeA: perfluorodecanoic acid; PFDoA: perfluorododecanoic acid; PFHpA: perfluoroheptanoic acid; PFHxA: perfluorohexanoic acid; PFHxS: perfluorohexane sulfonic acid; PFOA: perfluorooctanoic acid; PFNA: perfluorononanoic acid; PFOS: perfluorooctane sulfonic acid; PFOSA: perfluorooctane sulfonamide; PFPeA: perfluoropentanoic acid; PFUA: perfluoroundecanoic acid; PFUnDA: perfluoroundecanoic acid; PWV: pulse wave velocity; QGC: quantile g-computation; RCS: restricted cubic spline; SHAP: SHapley additive explanations; sICAM-1: soluble intercellular adhesion molecule-1; TC: total cholesterol; TNF-α: tumour necrosis factor-α; WQS: weighted quantile sum; XGBoost: extreme gradient boosting. Note: “ns” (not specified) is used in the Effect modifiers column for animal and in vitro studies because these controlled experimental studies do not require adjustment for confounding factors.

**Table 2:** Summary of studies investigating the associations between PFAS exposure and molecular biomarkers of vascular aging, including inflammatory, oxidative stress, epigenetic, proteomic, lipid-related, and endothelial dysfunction markers.

Main Findings	Effect Modifiers	Statistical Analysis	Molecular Biomarker	PFAS	Model/ Population	Study Type	First Author and Year
Oxidative stress, inflammation and endothelial dysfunction							
PFOS increased ROS and upregulated IL-1β, IL-6, COX-2, ICAM-1 and P-selectin, enhancing THP-1 adhesion.	ns	One-way ANOVA, Tukey test	Intracellular ROS, IL-1β, IL-6, COX-2, NOS3, ICAM-1, P-selectin, PPARγ, ERα, AHR	PFOS	HUVECs, THP-1 cells	In vitro	Liao et al., 2012 [37]
PFOS was associated with endothelial and platelet microparticles; elevated microparticles strengthened the association with increased CIMT (OR = 2.86, 95% CI 1.69–4.84).	Age, gender, smoking status, BMI, SBP, LDL-C, HDL-C, TG, HOMA-IR, hs-CRP.	Multiple linear and logistic regression	CD31+/CD42a− EMPs, CD31+/CD42a+ PMPs, HDL, LDL, 8-OHdG, CD62E, TG, CRP	PFOS PFOA PFCs	848 Taiwanese adolescents and young adults	Cross-sectional	Lin et al., 2016 [33]
Prenatal PFOS exposure increased blood pressure, impaired acetylcholine-induced relaxation, and reduced eNOS activation/expression.	ns	ANOVA, Dunnet’s post hoc test, unpaired Student’s *t*-test non-parametric Kruskal–Wallis test, Dunn’s multiple comparisons	eNOS, phospho-eNOS	PFOS	Offspring of PFOS-exposed pregnant rats	In vivo animal model	Dangudubiyyam et al., 2020 [41]
PFOS induced ferroptosis, increased lipid ROS levels and ACSL4 expression, and reduced GPX4, FTH1, HO-1 expression and NO content.	ns	ANOVA	Lipid ROS, NO, GPX4, ACSL4, FTH1, HO-1	PFOS	HUVECs	In vitro	Cui et al., 2022 [38]
PFOS promoted M1 macrophage polarization, thereby increasing TNF-α, IL-6, IL-1β, and iNOS expression, while suppressing M2 polarization by reducing CD206, Arg-1, and IL-10 promoting atherosclerosis in mice.	ns	ANOVA, LSD test, Dunnett’s test	TC, TG, LDL-C, HDL-C, NF-κB, iNOS, TNF-α, IL-6, IL-1β, CD206, Arg-1, IL-10	PFOS	ApoE^−/−^ mice and RAW264.7 macrophages	In vivo and in vitro	Wang et al., 2023 [25]
PFOS activated HRI/eIF2α/ATF4 ER-stress signaling and increased COX-2, ICAM-1, and IL-6 expression.	ns	ANOVA, Dunnett’s test	ATF4, C/EBPβ, COX-2, ICAM-1, IL-6, NF-κB, JAK2/STAT3	PFOS	HMEC-1 cells	In vitro and transcriptomics	Vajeethaveesin et al., 2025 [39]
F-53B plus nanoplastics activated NF-κB/NLRP3 signaling, increasing IL-1β, CASP1 and GSDMD, and inducing endothelial pyroptosis.	ns	ANOVA, LSD test, Dunnett’s test	NF-κB, NLRP3, ASC, CASP1, GSDMD, IL-1β, ICAM-1, VCAM-1	F-53B (6:2 Cl-PFESA)	C57BL/6J mice and HUVECs	In vivo and in vitro	Lv et al., 2025 [24]
OBS exposure → ROS accumulation, PERK–eIF2α–ATF4 ER stress and NF-κB activation; NAC and 4-PBA attenuated these effects. In vivo experiments: OBS exposure → endothelial impairment, collagen deposition, oxidative stress, ↑ ER stress markers, ↑ inflammation-related markers.	ns	ANOVA, unpaired Student’s *t*-test, Kruskal–Wallis test	ROS, PERK, IκBα, eIF2α, ATF4, NF-κB, ICAM-1, VCAM-1, IL-1β, IL-6, TNF-α, ZO-1, occludin, claudin-1, VE-cadherin	OBS	ApoE^−/−^ mice and HUVECs	In vivo and in vitro	Zhang et al., 2026 [40]
PFAS mixtures were associated with hypercholesterolemia (OR = 1.20), high LDL-C (OR = 1.13), hypertension (OR = 1.10) and hyperuricemia (OR = 1.31); both inflammatory markers mediated the associations.	Age, gender, residence, education, household income, tobacco, alcohol, physical activity, total energy intake, total fat intake, total cholesterol intake, BMI for non-obesity outcomes, BMI and serum creatinine for hyperuricemia	*t*-test, Wilcoxon, χ^2^ test, Spearman correlation analysis, multivariable linear/logistic regression, QGC and WQS mixture analyses, BKMR and RCS exposure, mediation analysis, FDR correction	hs-CRP, serum ferritin	PFAS mixture	8100 Chinese adult subjects	Cross-sectional	Zhao et al., 2026 [42]
Lipid metabolism dysregulation							
PFOS was positively associated with LDL-C and TC; PFNA, PFOS, and PFOA were associated with TG and AIP. PPAR signaling was identified as a core pathway.	Age, gender, race, family income-to-poverty ratio, albumin, uric acid, BMI, serum cotinine (tobacco exposure), diabetes, vitamin D	Weighted linear regression, Pearson correlation, BKMR, WQS, Mediation	LDL-C, TC, TG, AIP, serum folate, ALB, PPARγ, IL-10 (network), CASP1, CA2 (shared targets)	PFOA PFOS PFHxS PFNA	1099 adolescents aged 12–19 (US), enrolled in the 2005–2018 NHANES study	Cross-sectional	Pan et al., 2025 [48]
PFOS/PFOA increased lipid accumulation and induced PPARγ/Nrf2 signaling, IL-1β, PAI-2, MMP-1, and MMP-12 expression.	ns	Two-tailed unpaired *t*-test, one-way ANOVA, post hoc Dunnett, post hoc Tukey	PPARγ, Nrf2, IL-1β, PAI-2, COX-2, AKR1C3, MMP-1, MMP-12, total cholesterol	PFOS PFOA	Human U937-derived macrophages	In vitro	Connolly et al., 2025 [43]
PFAS increased total cholesterol (+10%), IDL (+25%) and LDL7 (+206%), while upregulating foam-cell-associated genes.	ns	*t*-test Mann–Whitney, Shapiro–Wilk, Brown-Forsythe, RNA-seq (edgeR), FDR Benjamini-Hochberg, GO enrichment (DAVID)	Total cholesterol, IDL, LDL subfractions, HDL, LDL7 (densest subfraction), oxLDL, Fabp4, Fasn (foam cell markers), Cxcl2, Cxcl17 (chemokines), Plin1, Plin5 (perilipins), Abca1, Abcg1 (cholesterol efflux)	PFAS mixture (PFOA, PFOS, PFNA, PFHxS, GenX)	40 LDLr^−/−^ mice	In vivo animal model	Roth et al., 2026 [45]
OBS induced more rapid dyslipidemia and stronger vascular inflammatory responses than PFOS. OBS induced stronger endothelial barrier disruption and inflammation than PFOS, with activation of NF-κB and MAPK/ERK pathways.	ns	Student’s *t*-test, one-way ANOVA, Kruskal–Wallis test	TG, TC, LDL-C, HDL-C, IL-6, TNF-α, IL-1β, NF-κB and MAPK/ERK signaling, ZO-1, occludin, claudin-5, VE-cadherin, endothelial permeability and LDH release	PFOS OBS	ApoE^−/−^ mice, HUVECs	In vivo and in vitro	Zhang et al., 2025 [46]
Toxicogenomic analyses linked PFAS exposure to lipid metabolism, inflammation, apoptosis, and JAK-STAT signaling pathways.	Age, gender ethnicity, smoking, and BMI	One-way ANOVA, BKMR, WQS, toxicogenomic pathway enrichment analyses	Hematologic and lipid-metabolism-related pathways	PFAS mixtures	2014 University students (CN), 120 C57BL/6J mice, toxicogenomics database	Epidemiological, in vivo, toxicogenomic analyses	Du et al., 2025 [47]
PPARγ and lipid metabolism pathways emerged among the principal mechanisms linking PFAS exposure to cardiovascular disease.	ns	PPI analysis, GO/KEGG; molecular docking	STAT3, MMP9, NFκB1, CASP3, AKT1, PPARγ	PFHpA PFOA PFNA PFDA	Network toxicology and molecular docking	In silico study	Chai et al., 2026 [44]
Epigenetic modulation							
PFAS exposure was associated with downregulation of miR-101-3p, miR-144-3p, and miR-19a-3p.	ns	EdgeR differential expression analysis, pairwise *t*-tests, FDR correction, 2−ΔΔCq analysis, IPA functional analysis, Fisher’s exact test	miR-101-3p, miR-144-3p, miR-19a-3p	PFOS PFHxS PFOA	292 exposed women in Sweden	Human observational study	Xu et al., 2020 [50]
PFAS exposure was associated with 435 differentially methylated CpG sites at birth and at 12 years of age.	Age, gender, household income, maternal race/ethnicity, maternal smoking during pregnancy, serum cotinine, cell-type composition.	GEE, interaction analysis, FDR correction, GO enrichment analysis, linear regression analyses, Spearman correlation analysis, *t*-test, χ^2^, Wilcoxon	CpG DNA methylation	PFOA PFOS PFNA PFHxS	HOME mother–child cohort	Longitudinal EWAS	Liu et al., 2022 [49]
PFOS was positively associated with 5mdC/dG, suggesting a potential contribution to arteriosclerosis through DNA methylation.	Age, gender, BMI, alcohol, hyperlipidemia, hypertension, diabetes, smoking, household income	Linear regression analysis, structural equation modelling (SEM)	Global DNA methylation (5mdC/dG)	PFOS	1425 Taiwanese participants	Cross-sectional	Lin et al., 2022 [32]
PPARα, and PPARγ were identified as core PFAS-CVD genes together with several PFAS-associated miRNAs.	ns	GO/KEGG enrichment, PPI analysis	PPARα, PPARγ, miR-130b-3p, miR-130a-3p, miR-129-5p	Multiple PFAS	Network toxicology	In silico study	Karakuş et al., 2024 [52]
PFAS exposure was associated with altered circulating miRNA profiles; miR-148b-3p and miR-29a-3p were consistently downregulated.	Age, BMI, race, weight loss prior to surgery, parents’ income, clinical site of surgery; Rhea Study: age, BMI, sex, parental education	Linear regression analysis, pathway enrichment	miR-148b-3p, miR-29a-3p	Multiple PFAS, PFAS mixture	176 Teen-LABs subjects (US), 64 Rhea Study subjects (GR)	Human cohort study	Li et al., 2024 [51]
OBS altered miRNA–mRNA networks associated with endothelial dysfunction and increased intracellular ROS.	ns	ANOVA, Tukey’s test, Kruskal–Wallis test	74 DEMs, 685 DEGs, miRNA–mRNA pairs, ROS	OBS	HUVECs	In vitro transcriptomic study	Zhang et al., 2026 [53]

Abbreviations: 6:2 Cl-PFESA (F-53B): 6:2 chlorinated polyfluoroalkyl ether sulfonic acid; 8-OHdG: 8-hydroxy-2′-deoxyguanosine; Abca1: ATP-binding cassette subfamily A member 1; Abcg1: ATP-binding cassette subfamily G member 1; ACSL4: Acyl-CoA synthetase long-chain family member 4; AHR: aryl hydrocarbon receptor; ALB: albumin; Arg-1: arginase-1; ANOVA: analysis of variance; ASC: apoptosis-associated speck-like protein containing a CARD; ATF4: activating transcription factor 4; BKMR: Bayesian kernel machine regression; BMI: body mass index; C/EBPβ: CCAAT/enhancer-binding protein beta; CD62E: E-selectin; CIMT: carotid intima–media thickness; COX-2: Cyclooxygenase-2; DEGs: differentially expressed genes; DEMs: differentially expressed miRNAs; DNA: Deoxyribonucleic acid; EMPs: endothelial microparticles; eNOS: endothelial nitric oxide synthase; ER: endoplasmic reticulum; ERα: estrogen receptor alpha; FDR: false discovery rate; FTH1: ferritin heavy chain 1; GenX: hexafluoropropylene oxide dimer acid (HFPO-DA); GPX4: Glutathione peroxidase 4; GSDMD: Gasdermin D; HDL: high-density lipoprotein; HDL-C: high-density lipoprotein cholesterol; HMEC-1: Human microvascular endothelial cell line-1; HO-1: heme oxygenase-1; HOMA-IR: homeostasis model assessment of insulin resistance; hs-CRP: high-sensitivity C-reactive protein; HUVECs: Human umbilical vein endothelial cells; IL: interleukin; IκBα: inhibitor of nuclear factor kappa B alpha; LDH: lactate dehydrogenase; LDL-C: low-density lipoprotein cholesterol; miRNA: microRNA; NF-κB: nuclear factor κappa-light-chain-enhancer of activated B cells; NHANES: National Health and Nutrition Examination Survey; NLRP3: NLR family pyrin domain containing 3; NO: nitric oxide; NOS3: nitric oxide synthase 3; ns: not specified; oxLDL: oxidized low-density lipoprotein; PAI-2: plasminogen activator inhibitor-2; PFAS: per- and polyfluoroalkyl substances; PFDA: perfluorodecanoic acid; PFHpA: perfluoroheptanoic acid; PFHxS: perfluorohexane sulfonic acid; PFNA: perfluorononanoic acid; PFOA: perfluorooctanoic acid; PFOS: perfluorooctane sulfonic acid; phospho-eNOS: phosphorylated endothelial nitric oxide synthase; Plin1: perilipin-1; Plin5: perilipin-5; PMPs: platelet microparticles; PPARα: peroxisome proliferator-activated receptor alpha; OBS: sodium p-perfluorononenoxybenzene sulfonate; QGC: quantile g-computation; RCS: restricted cubic spline; ROS: reactive oxygen species; TC: total cholesterol; TNF-α: tumour necrosis factor-α; VCAM-1, vascular cell adhesion molecule-1; ZO-1: zonula occludens-1. Note: “ns” (not specified) is used in the Effect modifiers column for animal and in vitro studies because these controlled experimental studies do not require adjustment for confounding factors.

## Data Availability

No new data was created or analyzed in this study. Data sharing is not applicable to this article.

## Supplementary Materials

The following supporting information can be downloaded at: https://www.mdpi.com/article/10.3390/ijms27136064/s1.

## Abbreviations

The following abbreviations are used in this manuscript:

AKR1C3	Aldo-keto reductase family 1 member C3
AKT1	RAC-alpha serine/threonine-protein kinase
AMS	Artery Measurement Software
ANOVA	Analysis of variance
ApoE	Apolipoprotein E
ATF4	Activating transcription factor 4
CA2	Carbonic anhydrase 2
CASP	Caspase
CD	Cluster of differentiation
CIMT	Carotid intima–media thickness
COX-2	Cyclooxygenase-2
CT	Computed tomography
CXCL	C-X-C motif chemokine ligand
CYP	Cytochrome P450
DNA	Deoxyribonucleic acid
DNMT3A	DNA methyltransferase 3 alpha
DXA	Dual-energy X-ray absorptiometry
eIF2α	Eukaryotic translation initiation factor 2α
eNOS	Endothelial nitric oxide synthase
ERK	Extracellular signal-regulated kinase
ERS	Endoplasmic reticulum stress
FMD	Flow-mediated dilation
GenX	hexafluoropropylene oxide dimer acid
HUVECs	Human umbilical vein endothelial cells
ICAM-1	Intercellular adhesion molecule-1
IL	Interleukin
IMT	Intima–media thickness
JAK	Janus Kinase
LDL-C	Low-density lipoprotein cholesterol
LDLr	Low-density lipoprotein receptor
MAPK	Mitogen-activated protein kinase
miRNA/miR	MicroRNA
MMP	Matrix metalloproteinase
NF-κB	Nuclear factor κappa-light-chain-enhancer of activated B cells
NHANES	National Health and Nutrition Examination Survey
NLR	Nod-like receptors
NLRP3	NLR family pyrin domain containing 3
PCA	Principal component analysis
PERK	Protein kinase R (PKR)-like endoplasmic reticulum kinase
PFAS	Per- and polyfluoroalkyl substances
PFDA	Perfluorodecanoic acid
PFDeA	Perfluorodecanoic acid
PFHpA	Perfluoroheptanoic acid
PFHxS	Perfluorohexanesulfonic acid
PFNA	Perfluorononanoic acid
PFOA	Perfluorooctanoic acid
PFOS	Perfluorooctane sulfonate
PIVUS	Prospective Investigation of the Vasculature in Uppsala Seniors
PPAR	Peroxisome proliferator-activated receptor
PWV	Pulse wave velocity
ROS	Reactive oxygen species
STAT	Signal transducer and activator of transcription

## References

[1] Brunn H., Arnold G., Körner W., Rippen G., Steinhäuser K.G., Valentin I. (2023). PFAS: Forever chemicals—Persistent, bioaccumulative and mobile. Reviewing the status and the need for their phase out and remediation of contaminated sites. Environ. Sci. Eur..

[2] Gaines L.G.T. (2023). Historical and current usage of per- and polyfluoroalkyl substances (PFAS): A literature review. Am. J. Ind. Med..

[3] Botelho J.C., Kato K., Wong L.Y., Calafat A.M. (2025). Per- and polyfluoroalkyl substances (PFAS) exposure in the U.S. population: NHANES 1999–March 2020. Environ. Res..

[4] Sunderland E.M., Hu X.C., Dassuncao C., Tokranov A.K., Wagner C.C., Allen J.G. (2019). A review of the pathways of human exposure to poly- and perfluoroalkyl substances (PFASs) and present understanding of health effects. J. Expo. Sci. Environ. Epidemiol..

[5] Jian J.M., Chen D., Han F.J., Guo Y., Zeng L., Lu X., Wang F. (2018). A short review on human exposure to and tissue distribution of per- and polyfluoroalkyl substances (PFASs). Sci. Total Environ..

[6] Rosato I., Bonato T., Fletcher T., Batzella E., Canova C. (2024). Estimation of per- and polyfluoroalkyl substances (PFAS) half-lives in human studies: A systematic review and meta-analysis. Environ. Res..

[7] Fenton S.E., Ducatman A., Boobis A., DeWitt J.C., Lau C., Ng C., Smith J.S., Roberts S.M. (2021). Per- and polyfluoroalkyl substance toxicity and human health review: Current state of knowledge and strategies for informing future research. Environ. Toxicol. Chem..

[8] Ferguson E.J., Tessmann J.W., Zaytseva Y.Y. (2026). Impact of PFAS exposure on lipid metabolic pathways: Mechanisms and implicatins in carcinogenesis. Front. Toxicol..

[9] Dameris L., Carberry V., Seifert C., Sherman J., Hu Y., Rebuli M.E., Turpin B.J., Surratt J.D., Smith G., Rager J.E. (2026). Current status of per- and poly-fluoroalkyl substances (PFAS) exposure on lung cell biology and pulmonary outcomes along human health risk assessment steps. Curr. Allergy Asthma Rep..

[10] Yang X., Li X., Li X., Zhang H., Wang C., Chen X. (2026). Per- and polyfluoroalkyl substances and cardiovascular disease: A mechanistic and epidemiological synthesis. Ecotoxicol. Environ. Saf..

[11] Wen Z.J., Wei Y.J., Zhang Y.F., Zhang Y.F. (2023). A review of cardiovascular effects and underlying mechanisms of legacy and emerging per- and polyfluoroalkyl substances (PFAS). Arch. Toxicol..

[12] Meneguzzi A., Fava C., Castelli M., Minuz P. (2021). Exposure to perfluoroalkyl chemicals and cardiovascular disease: Experimental and epidemiological evidence. Front. Endocrinol..

[13] Lind P.M., Salihovic S., Stubleski J., Kärrman A., Lind L. (2018). Changes in plasma levels of perfluoroalkyl substances (PFASs) are related to increase in carotid intima-media thickness over 10 years—A longitudinal study. Environ. Health.

[14] Chen W., Qiu C., Yang Z., Nash H., Karim R., Chen Z. (2026). Per- and polyfluoroalkyl substances: A novel risk factor for cardiovascular disease. Ecotoxicol. Environ. Saf..

[15] Wittkopp S., Wu F., Windheim J., Robinson M., Kannan K., Katz S.D., Chen Y., Newman J.D. (2022). Vascular endothelium as a target for perfluoroalkyl substances (PFAS). Environ. Res..

[16] Osorio-Yáñez C., Sanchez-Guerra M., Cardenas A., Lin P.D., Hauser R., Gold D.R., Kleinman K.P., Hivert M.F., Fleisch A.F., Calafat A.M. (2021). Per- and polyfluoroalkyl substances and calcifications of the coronary and aortic arteries in adults with prediabetes: Results from the diabetes prevention program outcomes study. Environ. Int..

[17] Wang X., Cao Y., Chen X., Jian G., Ma F., Zhang H., Wang Q., Xiao W. (2025). Per- and polyfluoroalkyl substances (PFAS) exposure and cardiovascular risk: Lipid profile as a mediator. Lipids Health Dis..

[18] Dunder L., Salihovic S., Lind P.M., Elmståhl S., Lind L. (2023). Plasma levels of per- and polyfluoroalkyl substances (PFAS) are associated with altered levels of proteins previously linked to inflammation, metabolism and cardiovascular disease. Environ. Int..

[19] Gorini F., Tonacci A., Palazzo M., Bustaffa E., Minichilli F., Borghini A. (2026). Per- and polyfluoroalkyl substances exposure and ischemic heart disease: Emerging evidence from the literature. Antioxidants.

[20] Brieger A., Bienefeld N., Hasan R., Goerlich R., Haase H. (2011). Impact of perfluorooctanesulfonate and perfluorooctanoic acid on human peripheral leukocytes. Toxicol. In Vitro.

[21] Qazi M.R., Bogdanska J., Butenhoff J.L., Nelson B.D., DePierre J.W., Abedi-Valugerdi M. (2009). High-dose, short-term exposure of mice to perfluorooctanesulfonate (PFOS) or perfluorooctanoate (PFOA) affects the number of circulating neutrophils differently, but enhances the inflammatory responses of macrophages to lipopolysaccharide (LPS) in a similar fashion. Toxicology.

[22] Zota A.R., Geller R.J., Romano L.E., Coleman-Phox K., Adler N.E., Parry E., Wang M., Park J.S., Elmi A.F., Laraia B.A. (2018). Association between persistent endocrine-disrupting chemicals (PBDEs, OH-PBDEs, PCBs, and PFASs) and biomarkers of inflammation and cellular aging during pregnancy and postpartum. Environ. Int..

[23] Omoike O.E., Pack R.P., Mamudu H.M., Liu Y., Strasser S., Zheng S., Okoro J., Wang L. (2021). Association between per- and polyfluoroalkyl substances and markers of inflammation and oxidative stress. Environ. Res..

[24] Lv J., Tan Z., An Z., Xu R., Zhang H., Guo M., Xiao F., Zhao M., Guo Y., Liu Y. (2025). Co-exposure to polystyrene nanoplastics and F-53B induces vascular endothelial cell pyroptosis through the NF-κB/NLRP3 pathway. J. Hazard. Mater..

[25] Wang D., Tan Z., Yang J., Li L., Li H., Zhang H., Liu H., Liu Y., Wang L., Li Q. (2023). Perfluorooctane sulfonate promotes atherosclerosis by modulating M1 polarization of macrophages through the NF-κB pathway. Ecotoxicol. Environ. Saf..

[26] Wang J., Du B., Wu Y., Li Z., Wang H., Niu Y., Ye Y., Chen Q., Wang Q., Wu Y. (2023). Maternal plasma perfluoroalkyl substances concentrations in early pregnancy and cardiovascular development in offspring: A prospective cohort study. Environ. Int..

[27] Gump B.B., Hill D.T., Robinson M., Kannan K., Heffernan K., Atallah-Yunes N.H., Brann L., Parsons P.J., Palmer C.D., MacKenzie J.A. (2023). Perfluoroalkyl substances (PFAS) and lead (Pb) as “cardiovascular disruptors” in 9–11-year-old children living in Syracuse, New York, United States. Environ. Res..

[28] Lind P.M., Salihovic S., van Bavel B., Lind L. (2017). Circulating levels of perfluoroalkyl substances (PFASs) and carotid artery atherosclerosis. Environ. Res..

[29] Yang J., Wang T., Li K., Wang Y. (2025). Associations between per- and polyfluoroalkyl chemicals and abdominal aortic calcification in middle-aged and older adults. J. Adv. Res..

[30] Koskela A., Ducatman A., Schousboe J.T., Nahhas R.W., Khalil N. (2022). Perfluoroalkyl substances and abdominal aortic calcification. J. Occup. Environ. Med..

[31] Khalil N., Ducatman A.M., Sinari S., Billheimer D., Hu C., Littau S., Burgess J.L. (2020). Per- and polyfluoroalkyl substance and cardiometabolic markers in firefighters. J. Occup. Environ. Med..

[32] Lin C.Y., Lee H.L., Chen C.W., Wang C., Sung F.C., Su T.C. (2022). Global DNA methylation mediates the association between serum perfluorooctane sulfonate and carotid intima-media thickness in young and middle-aged Taiwanese populations. Ecotoxicol. Environ. Saf..

[33] Lin C.Y., Chen P.C., Lo S.C., Torng P.L., Sung F.C., Su T.C. (2016). The association of carotid intima-media thickness with serum level of perfluorinated chemicals and endothelium-platelet microparticles in adolescents and young adults. Environ. Int..

[34] Lin C.Y., Lin L.Y., Wen T.W., Lien G.W., Chien K.L., Hsu S.H., Liao C.C., Sung F.C., Chen P.C., Su T.C. (2013). Association between levels of serum perfluorooctane sulfate and carotid artery intima-media thickness in adolescents and young adults. Int. J. Cardiol..

[35] Liberda E.N., Zuk A.M., Tsuji L.J.S. (2019). Complex contaminant mixtures and their associations with intima-media thickness. BMC Cardiovasc. Disord..

[36] Li H., Yang M., Zhao J., Tan Z., Li L., An Z., Liu Y., Liu X., Zhang X., Lu J. (2024). Association of per- and polyfluoroalkyl substance exposure with coronary stenosis and prognosis in acute coronary syndrome. Environ. Health.

[37] Liao Y., Wang J., Huang Q.S., Fang C., Kiyama R., Shen H., Dong S. (2012). Evaluation of cellular response to perfluorooctane sulfonate in human umbilical vein endothelial cells. Toxicol. In Vitro.

[38] Cui J., Wang P., Yan S., Liang Y., Liu D., Ren S. (2022). Perfluorooctane sulfonate induces dysfunction of human umbilical vein endothelial cells via ferroptosis pathway. Toxics.

[39] Vajeethaveesin N., Kanitwithayanun J., Suriyo T., Chujan S., Satayavivad J. (2025). Perfluorooctane sulfonic acid: A possible risk factor of endothelial dysfunction based on in silico and in vitro studies. Arch. Toxicol..

[40] Zhang B., Liu Z., Li Q., Tian M., Gao Y., Kishi H., Xu D. (2026). Endoplasmic reticulum stress mediates oxidative stress-driven endothelial impairment and atherogenesis induced by sodium perfluorononenoxybenzene sulfonate exposure. Environ. Res..

[41] Dangudubiyyam S.V., Mishra J.S., Zhao H., Kumar S. (2020). Perfluorooctane sulfonic acid (PFOS) exposure during pregnancy increases blood pressure and impairs vascular relaxation mechanisms in the adult offspring. Reprod. Toxicol..

[42] Zhao Y., Wang H., Hu Y., Li Z., Kang X., Su C., Wu Z., Zhang T., Liu A. (2026). Serum perfluoroalkyl and polyfluoroalkyl substances and the risk of cardiometabolic diseases: Unraveling the mediating role of inflammatory markers. Eco-Environ. Health.

[43] Connolly J.C., Ishihara Y., Sawaya E., Whitfield V., Garrity N., Sohata R., Tsymbal M., Lundberg A., La Merrill M.A., DeWitt J.C. (2025). Per- and polyfluoroalkyl substances (PFAS) enhance cholesterol accumulation and dysregulate inflammatory responses in macrophages. Cardiovasc. Toxicol..

[44] Chai J., Wang Y., Zhang C., Wang Y., Xue A., Jie W. (2026). Mechanistic insights into PFAS derivatives-induced coronary heart disease and atherosclerotic renal artery stenosis via integrated network toxicology and molecular modeling. Toxicol. Res..

[45] Roth K., Yang Z., Agarwal M., Gurdziel K., Petriello M.C. (2026). Exposure to a PFAS mixture alters cholesterol lipoprotein subfractions and induces a foam cell-like aortic macrophage expression profile in hyperlipidemic LDLr^−/−^ mice. Toxicol. Appl. Pharmacol..

[46] Zhang B., Li Q., Wang W., Tian M., Xu D., Xie Y. (2025). PFOS and its substitute OBS cause endothelial dysfunction to promote atherogenesis in ApoE^−/−^ mice. Environ. Health.

[47] Du X., Xu X., Dong X.X., Liang X., Wu Y., Du Z., Pan C.W., Liang G., Li Y.Z., Zheng Y.J. (2025). Integration of animal, population, and toxicogenomic evidence on the hematotoxic and immunosuppressive effects of environmental exposure to PFAS mixtures in adolescents. Environ. Sci. Technol..

[48] Pan Y., Du Z., Ma Y., Chen C., He S., Zhang M., Baral K., Xu L., Xu M., Zhao M. (2025). Suppression effect of folate on poly- and perfluoroalkyl substance-induced alterations in lipids and the atherogenic index of plasma in adolescents. Lipids Health Dis..

[49] Liu Y., Eliot M.N., Papandonatos G.D., Kelsey K.T., Fore R., Langevin S., Buckley J., Chen A., Lanphear B.P., Cecil K.M. (2022). Gestational perfluoroalkyl substance exposure and DNA methylation at birth and 12 years of age: A longitudinal epigenome-wide association study. Environ. Health Perspect..

[50] Xu Y., Jurkovic-Mlakar S., Li Y., Wahlberg K., Scott K., Pineda D., Lindh C.H., Jakobsson K., Engström K. (2020). Association between serum concentrations of perfluoroalkyl substances (PFAS) and expression of serum microRNAs in a cohort highly exposed to PFAS from drinking water. Environ. Int..

[51] Li Y., Baumert B.O., Stratakis N., Goodrich J.A., Wu H., Liu S.H., Wang H., Beglarian E., Bartell S.M., Eckel S.P. (2024). Exposure to per- and polyfluoroalkyl substances and alterations in plasma microRNA profiles in children. Environ. Res..

[52] Karakuş F., Kuzu B. (2024). Predicting the molecular mechanisms of cardiovascular toxicity induced by per- and polyfluoroalkyl substances: An in silico network toxicology perspective. Toxicol. Res..

[53] Zhang B., Li Q., Ding X., Tian M., Xu D. (2026). Exposure to OBS induces endothelial dysfunction via miRNA–mRNA regulatory networks: Implications for cardiovascular disease risk. Comp. Biochem. Physiol. C Toxicol. Pharmacol..

