# Supplementary material for: Imaging and Molecular Biomarkers of PFAS-Related Vascular Aging: A Narrative Review
Source: Int J Mol Sci. 2026 Jul 6;27(13):6064. doi: 10.3390/ijms27136064 (PMC13361577; doi:10.3390/ijms27136064)
Supplement: Supplementary file 1 [file ijms-27-06064-s001.zip › ijms-4400716-supplementary.pdf]

Supplementary Materials

For imaging biomarkers, the following search strategies were applied:

PubMed: (PFAS OR "perfluoroalkyl substances" OR "polyfluoroalkyl substances" OR PFOA OR PFOS) AND ("vascular aging" OR "arterial stiffness" OR "pulse wave velocity" OR PWV OR cfPWV OR "intima-media thickness" OR IMT OR CIMT OR "endothelial function" OR "flow-mediated dilation" OR FMD OR calcification OR "coronary stenosis").

Scopus: TITLE-ABS-KEY ((PFAS OR "perfluoroalkyl substances" OR "polyfluoroalkyl substances" OR PFOA OR PFOS) AND ("vascular aging" OR "arterial stiffness" OR "pulse wave velocity" OR PWV OR cfPWV OR "intima-media thickness" OR IMT OR CIMT OR "endothelial function" OR "flow-mediated dilation" OR FMD OR calcification OR "coronary stenosis")).

For molecular biomarkers, the following search strategies were applied:

PubMed: ("PFAS"[Title/Abstract] OR "perfluoroalkyl substances"[Title/Abstract] OR "polyfluoroalkyl substances"[Title/Abstract] OR "PFOA"[Title/Abstract] OR "PFOS"[Title/Abstract]) AND ("Oxidative Stress"[MeSH Terms] OR "Oxidative Stress"[Title/Abstract] OR "redox imbalance"[Title/Abstract] OR "ROS"[Title/Abstract] OR "Inflammation"[MeSH Terms] OR "Inflammation"[Title/Abstract] OR "mitochondrial damage"[Title/Abstract] OR "endothelial dysfunction"[Title/Abstract] OR "endothelial damage"[Title/Abstract] OR "DNA Damage"[MeSH Terms] OR "DNA damage"[Title/Abstract] OR "DNA strand break"[Title/Abstract] OR "genotoxicity"[Title/Abstract] OR "telomere"[Title/Abstract] OR "telomere length"[Title/Abstract] OR "telomere shortening"[Title/Abstract] OR "Epigenetic, Genetic"[MeSH Terms] OR "epigenetic"[Title/Abstract] OR "DNA methylation"[Title/Abstract] OR "miRNA"[Title/Abstract] OR "microRNA"[Title/Abstract] OR "histone acetylation"[Title/Abstract] OR "acetylation"[Title/Abstract] OR "DNA copy number"[Title/Abstract] OR "copy number variation"[Title/Abstract] OR "CNV"[Title/Abstract] OR "dyslipidemia"[MeSH Terms] OR "dyslipidemia"[Title/Abstract] OR "lipid metabolism"[Title/Abstract] OR "lipid profile"[Title/Abstract] OR "hyperlipidemia"[Title/Abstract] OR "hypercholesterolemia"[Title/Abstract] OR "LDL"[Title/Abstract] OR "HDL"[Title/Abstract] OR "triglycerides"[Title/Abstract]) AND ("Cardiovascular Diseases"[MeSH Terms] OR "Atherosclerosis"[MeSH Terms] OR "vascular disease"[Title/Abstract] OR "vascular aging"[Title/Abstract] OR "cardiovascular disease"[Title/Abstract] OR "atherosclerosis"[Title/Abstract]).

Scopus: TITLE-ABS-KEY ("PFAS" OR "perfluoroalkyl substances" OR "polyfluoroalkyl substances" OR "PFOA" OR "PFOS") AND TITLE-ABS-KEY ("oxidative stress" OR "redox imbalance" OR "ROS" OR "inflammation" OR "mitochondrial damage" OR "endothelial dysfunction" OR "endothelial damage" OR "DNA damage" OR "DNA strand break" OR "genotoxicity" OR "telomere" OR "telomere length" OR "telomere shortening" OR "epigenetic" OR "epigenetics" OR "DNA methylation" OR "miRNA" OR "microRNA" OR "histone acetylation" OR "acetylation" OR "DNA copy number" OR "copy number variation" OR "CNV" OR "dyslipidemia" OR "lipid metabolism" OR "lipid profile" OR "hyperlipidemia" OR "hypercholesterolemia" OR "LDL" OR "HDL" OR "triglycerides") AND TITLE-ABS-KEY ("vascular disease" OR "vascular aging" OR "cardiovascular disease" OR "atherosclerosis").
